# Characterization and standardization of tissue-simulating protoporphyrin IX optical phantoms

**DOI:** 10.1117/1.JBO.21.3.035003

**Published:** 2016-03-11

**Authors:** Mikael Marois, Jaime Bravo, Scott C. Davis, Stephen Chad Kanick

**Affiliations:** a Polytechnique Montreal, 2500 Chemin de Polytechnique, Montreal, Quebec H3T 1J4, Canada; b Dartmouth College, 14 Engineering Drive, Hanover, New Hampshire 03755, United States; c Norris Cotton Cancer Center, Dartmouth Hitchcock Medical Center, 1 Medical Center Drive, Lebanon, New Hampshire 03766, United States

**Keywords:** tissue-simulating phantoms, fluorescence spectroscopy, optical properties

## Abstract

Optical devices for measuring protoporphryin IX (PpIX) fluorescence in tissue are routinely validated by measurements in optical phantoms. Yet there exists limited data to form a consensus on the recipe for phantoms that both mimic the optical properties found in tissue and yield a reliable and stable relationship between PpIX concentration and the fluorescence remission intensity. This study characterizes the influence of multiple phantom components on PpIX fluorescence emission intensity, using Intralipid as the scattering source, bovine whole blood as the background absorber, and Tween as a surfactant to prevent PpIX aggregation. Optical measurements showed a linear proportionality (r>0.99) between fluorescence intensity and PpIX concentration (0.1 to 10  μg/mL) over a range of Intralipid (1 to 2%) and whole blood (0.5 to 3%) for phantoms containing low surfactant (≤0.1%), with fluorescence intensities and scattering and absorption properties stable for 5 h after mixing. The role of surfactant in PpIX phantoms was found to be complex, as aggregation was evident in aqueous nonturbid phantoms with no surfactant (0% Tween), and avoided in phantoms containing Intralipid as the scattering source with no additional or low amounts of added surfactant (≤0.1% Tween). Conversely, phantoms containing higher surfactant content (>0.1% Tween) and whole blood showed interactions that distorted the fluorescence emissions.

## 1 Introduction

Protoporphyrin IX (PpIX) is a photosensitizer and fluorophore of interest both for experimental photo-based therapies and for diagnostic assessment of suspicious tissue during surgery.^1^ While PpIX occurs naturally in the body, temporary enhancement can be induced by administration of aminolevulinic acid (ALA), a nonfluorescent prodrug that serves to bypass the negative feedback controls of the heme-biosynthesis pathway and results in a selective retention of PpIX in tissue with altered metabolic activity.^2^^,^^3^ In the field of dermatology, topical application of ALA induces PpIX in precancerous and cancerous skin lesions, where PpIX serves as a photosensitizer for photodynamic therapy.^4^^,^^5^ Dosimetry studies have shown that optical measurements of PpIX fluorescence may provide insight into patient-specific responses to therapy.^6^^–^^8^ In the field of neurosurgery, oral administration of ALA is used to induce PpIX accumulation in malignant tumors in the brain.^2^ Multiple clinical investigations are considering the use of optical measurements of PpIX fluorescence to increase contrast between tumor and surrounding normal cortex.^9^^–^^14^ Research in these clinical specialties has led to the development of a variety of approaches to optically sample PpIX fluorescence emissions from tissue.^1^^,^^15^ Basic imaging approaches sample raw PpIX fluorescence intensity resulting from wide-field illumination with the excitation wavelength,^7^^,^^9^^,^^16^ which provides a relative evaluation of PpIX concentration changes within a field of view. Spectroscopic approaches are able to un-mix fluorescence emissions originating from both PpIX and endogenous fluorochromes^8^^,^^17^^–^^20^ and correct for distortions from background optical properties,^8^^,^^17^^–^^21^ with many of these approaches returning quantitative metrics of PpIX fluorescence that are comparable between different measurement locations. One requirement shared by all of these optical measurement devices is that the sampled fluorescence intensity attributed to PpIX be proportional to PpIX concentration. This validation step is often performed using measurements of tissue-simulating optical phantoms.^8^^,^^16^^,^^20^^,^^22^^–^^25^ Such benchtop optical phantom studies are used to determine the lower limits of detection of PpIX in turbid media, and also are used to assess the sensitivity of collected fluorescence emissions to variations in background optical properties.

**Fig. 1:**
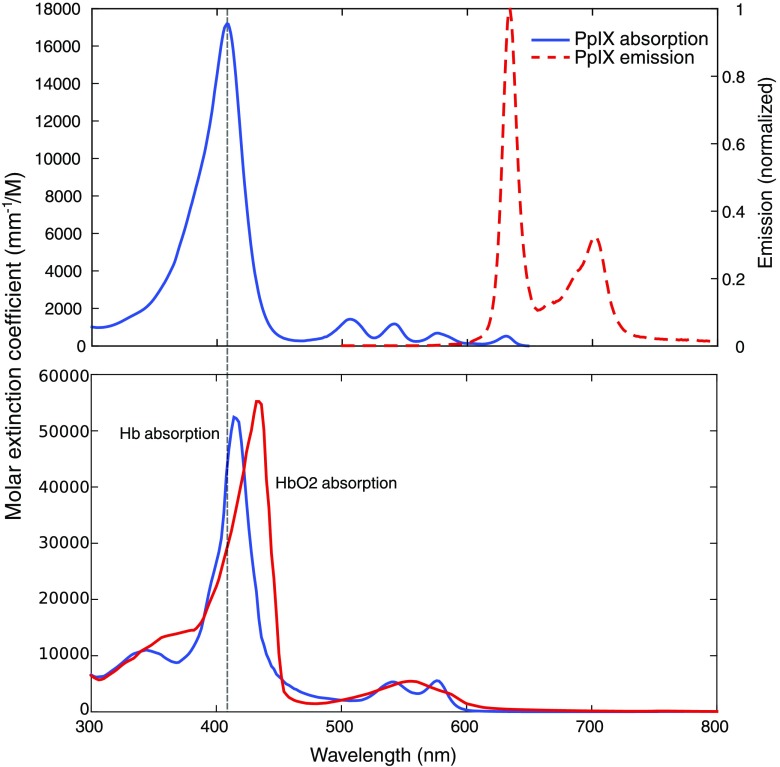
Absorption and emission of PpIX compared to absorption of Hb and ${\mathrm{HbO}}_{2}$. A horizontal line at 405 nm highlights a commonly used excitation wavelength to measure PpIX fluorescence.

In order for measurements in optical phantoms to be representative of measurements in tissue, the phantoms need to mimic the wavelength-dependent optical properties endogenous to tissue. Figure 1 shows the absorption and emission spectra of PpIX, as well as the absorption coefficient of oxygenated and deoxygenated hemoglobin, which are dominant endogenous absorbers in tissue. PpIX is commonly excited in the blue wavelength region, where a strong absorption band at 405 nm yields efficient fluorescence generation. However, blue excitation competes with background absorption from the soret band of hemoglobin, resulting in a tissue-surface-weighted estimate of PpIX. Methods that excite PpIX in the red (near 633 nm) yield a less efficient fluorescent signal but avoid the strong hemoglobin-based attenuation and achieve deeper sampling. Previously reported studies designed to validate PpIX quantification systems have used optical phantoms with components that closely match the wavelength dependence of absorption and scattering observed in tissue,^8^^,^^16^^,^^20^^,^^22^^–^^25^ including the use of Intralipid as a scattering source and whole blood as an absorber. While these components are not recognized as optical property standards due to a lack of quality control, they have been extensively characterized to provide combinations of wavelength-dependent scattering^26^^–^^28^ and absorption^29^ properties in the excitation and emission bands for PpIX that closely mimic tissue. An additional, yet critical, aspect to consider is the hydrophobicity of PpIX, which can lead to aggregation and decreased fluorescence when placed into an aqueous solution.^30^^,^^31^ To mitigate this effect, surfactants such as polyethylene glycol sorbitan esters (e.g., Tween), are commonly added to aqueous PpIX phantoms to prevent aggregation.^8^^,^^20^^,^^22^^–^^25^ However, surfactants are also known to lyse or otherwise affect blood cells,^32^ which in turn can impact the optical properties of the phantom solution. Yet, to date, a methodical study to examine interactions of constituents and optical property stability in PpIX phantoms has not been reported.

This study aims to identify an optimal and standardized PpIX phantom recipe for instrument validation and performance assessment studies. Toward that end, the study considers how different recipes for PpIX tissue-simulating optical phantoms influence the proportionality between sampled fluorescence and the PpIX concentration contained within the phantom. The study highlights how certain combinations of the basic recipe components can have profound and unintended influences on collected PpIX fluorescence emissions. The presented data informs the standardization of the phantom making process for tissue-mimicking PpIX phantoms for fluorescence detection systems.

## 2 Materials and Methods

Tissue-simulating PpIX phantoms created using various recipes were used to characterize the effects of phantom composition on fluorescence emission spectra. The optical phantoms were measured using either a spectrofluorometer for nonscattering solutions, or with a fiber-optic-based spectroscopy system for phantoms containing a scattering agent.

### 2.1 Optical Phantom Preparation

Nonturbid (i.e., clear) phantoms were prepared to characterize the fluorescence emission of PpIX in an aqueous solution in the presence of various amounts of surfactant. For this set, 15 phantoms (10 mL each) were prepared with PpIX (Sigma-Aldrich, St. Louis, Missouri) solubilized in dimethyl sulfoxide (DMSO) to yield concentrations of (0.1, 1, 10) μg/mL, Tween 20 (Sigma-Aldrich) in volume fractions (TVF) of (0, 0.1, 0.5, 1, 5)%, and the balance completed with phosphate-buffered saline (PBS). An additional phantom set was also measured using egg yolk (more specifically the amphipathic phospholipid contents of egg yolk^33^^,^^34^) as a surfactant, with egg yolk volume fractions of (0, 0.1, 0.5, 1, 5)%.

Two phantom sets were constructed to characterize PpIX fluorescence in turbid phantoms in the presence of surfactant: one set containing Intralipid 20% (Fresenius Kabi, Uppsala, Sweden) as the scattering agent with no background absorber, and a second set containing both Intralipid and bovine whole blood (Lampire, Ottsville, *Pennsylvania*) as a background absorber. Each phantom in this set contained 1.5% lipid volume fraction (LVF), while the absorbing set of phantoms also contained 2% blood volume fraction (BVF). PpIX was solubilized using DMSO at a stock concentration of 1  mg/mL; dilutions from this stock were used to achieve the desired PpIX concentration in each phantom. In total, 50 phantoms (20 mL each) were constructed to include a range of five PpIX concentrations [(0.1, 0.3, 1, 3, 10) μg/mL], each prepared with five different TVF [(0, 0.1, 0.5, 1, 5)%]. Additionally, a phantom set with 1.5% LVF, 2% BVF, and 1 μg/mL PpIX was sampled with a more granular set of TVF values [(0, 0.1, 0.5, 1, 2, 3, 4, 5)%]. To confirm the stability and reliability of Intralipid as a scatterer and blood as an absorber for fluorescence measurements, two additional sets of phantoms were made varying LVF and BVF at a TVF of 0.1% for a range of PpIX concentrations [(0.1, 0.3, 1, 3 10) μg/mL]. This included variation of BVF [(0.5, 1, 2, 3)%] with a fixed LVF of 1.5%, and also variation of LVF [(1, 1.5, 2)%] with a fixed BVF of 2%. Table 1 provides a detailed overview of the combinations of constituents used within each of the phantom sets.

**Table 1:** Detailed list of components used in the phantom sets.

Phantom set	PpIX (μg/mL)	LVF (%)	BVF (%)	TVF (%)	Egg yolk (%)
Nonturbid—TVF	0.1, 1, 10	0	0	0, 0.1, 0.5 1, 5	0
Nonturbid—Egg yolk	0.1, 1, 10	0	0	0	0, 0.1, 0.5, 1, 5
Turbid—TVF #1	0.1, 0.3, 1, 3, 10	1.5	2	0, 0.1, 0.5, 1, 5	0
Turbid—TVF #2	1	1.5	2	0, 0.1, 0.5, 1, 2, 3, 4, 5	0
Turbid—BVF	0.1, 0.3, 1, 3, 10	1.5	0.5, 1, 2, 3	0.1	0
Turbid—LVF	0.1, 0.3, 1, 3, 10	1, 1.5, 2	2	0.1	0

### 2.2 Spectroscopic Equipment

Nonturbid optical phantoms were measured using the FluoroMax-3 spectrofluorometer (HORIBA Scientific, Edison, New Jersey). During the measurements, excitation light at 405 nm was projected on the samples, and fluorescence emissions were collected over the range of (600 to 750) nm (Δλ=1  nm) at an angle perpendicular to excitation light. Turbid optical phantoms were measured using a custom-built fiberoptic probe system described in detail previously.^18^ The system included a hand-held probe that contained four active fiber optic leads of 200  μm in diameter: a detector fiber [connected to a spectrophotometer (USB2000+, Ocean Optics, Dunedin, Florida)], a blue light-emitting diode (LED) (405 nm) source (LedEngin Inc. Santa Clara, California) fiber with a center to center separation from the detector of 260  μm, and two fibers that lead to a white LED light source (LedEngin Inc. Santa Clara, California) spaced 260 and 520  μm center-to-center from the detector. The four fiber-optic leads were connected to a multiplexer (Model MPM-2000, Ocean Optics, Dunedin, Florida) such that the detected light could be measured sequentially by the spectrophotometer. The probe was controlled using LabVIEW (National Instruments, Austin, Texas) and measurements were taken with the probe tip placed few millimeters beneath the surface of the liquid phantom.

### 2.3 Data Sampling

Optical measurements were performed in the phantoms immediately after components were mixed and sampling was repeated every hour for 5 h. All phantoms were stored in the dark between measurements to prevent PpIX photobleaching from ambient light and mixed before measurement by inverting the sample multiple times to re-mix constituents and prevent blood pooling prior to each optical measurement. All phantoms were stored at ambient room temperature (≈21°C) except for two additional phantoms [LVF = 1.5%, BVF = 2%, PpIX = 1 μg/mL, TVF = (0.5, 5)%] that were stored in a refrigeration unit (≈2°C) during the 5-h period. Each optical measurement consisted of a paired measurement of reflectance, using white light, and fluorescence, using blue light. Optical measurements were repeated in each phantom multiple times.

#### 2.3.1 Reflectance fitting

Raw reflectance spectra measured from a sample was calibrated by subtracting the dark current, dividing by integration time, and then normalizing by a ratio of the model-estimated and measured white light reflectance spectra of an Intralipid reference phantom containing 2% LVF. Model estimates for the calibration reference was calculated using the optical properties in Intralipid reported previously,^26^ and constrained diffusion theory was used as the inversion technique between spectra and optical properties within this study. Work by Kim et. al^35^ details the mathematical model for multi-fiber applications, which returns an estimate of diffuse reflectance using estimates of the absorption coefficient ($\mu_{a}$), the reduced scattering ($\mu_{s}^{\prime }$), and the source-detector separation (ρ) as inputs such that $R(\lambda )=f(\mu_{a}(\lambda ),\mu_{s}^{\prime }(\lambda ),\rho )$. $\mu_{s}^{\prime }(\lambda )$ was estimated using a wavelength dependent power law relationship,^36^ as

$$\mu_{s}^{\prime }(\lambda )=\mu_{s}^{\prime }(\lambda_{o}){\left(\frac{\lambda }{\lambda_{o}}\right)}^{-b},$$ (1)

where $\mu_{s}^{\prime }(\lambda_{o})$ and b, the scattering slope, are fitted values and $\lambda_{o}$ was set to 550 nm. $\mu_{a}(\lambda )$ was modeled as a linear sum of all significant chromophores, as

$$\mu_{a}(\lambda )=\mathrm{BVF}[{\mathrm{StO}}_{2}\epsilon_{a}^{\mathrm{oxyHb}}(\lambda )+(1-{\mathrm{StO}}_{2})\epsilon_{a}^{\mathrm{deoxyHb}}(\lambda )],$$ (2)

where, BVF is the blood volume fraction, ${\mathrm{StO}}_{2}$ is the microvascular saturation, and $\epsilon_{a}^{\mathrm{oxyHb}}$ and $\epsilon_{a}^{\mathrm{deoxyHb}}$ are the wavelength-dependent specific absorption coefficients (${\mathrm{cm}}^{-1}$) of fully oxygenated and deoxygenated hemoglobin, respectively. Reflectance fits were performed over the wavelength range 450–650 nm, with parameters estimated in the set: [BVF, ${\mathrm{StO}}_{2}$, $\mu_{s}^{\prime }(\lambda_{o})$, and b].

#### 2.3.2 Fluorescence fitting

Fluorescence spectra were calibrated by subtracting the dark current and dividing by integration time before fitting the resulting spectra as a linear combination of emission profiles from PpIX, PpIX photoproducts, and autofluorescence emission, as

$$F_{\text{Raw}}(\lambda )=[{\mathrm{FL}}^{\mathrm{PpIX}}\epsilon_{e}^{\mathrm{PpIX}}+\Sigma ({\mathrm{FL}}^{\mathrm{PP}}\epsilon_{e}^{\mathrm{PP}})+{\mathrm{FL}}^{\mathrm{AF}}\epsilon_{e}^{\mathrm{AF}}],$$ (3)

where $\epsilon_{e}^{\mathrm{PpIX}}$, $\epsilon_{e}^{\mathrm{PP}}$, and $\epsilon_{e}^{\mathrm{AF}}$ are normalized wavelength-dependent emission profiles and ${\mathrm{FL}}^{\mathrm{PpIX}}$, ${\mathrm{FL}}^{\mathrm{PP}}$, and ${\mathrm{FL}}^{\mathrm{AF}}$ are fitted estimates of the fluorescence contribution [a.u.] of PpIX, PpIX’s photoproducts, and autofluorescence, respectively. Estimates obtained using Eq. (3) are influenced by fluorescence distortions due to optical properties. To correct for these distortions, we utilized a fluorescence quantification algorithm described by Kim et. al.^18^ used to extract intrinsic fluorescence ($F_{\text{Quant}}$) from a paired measurement of reflectance and fluorescence, such that

$$F_{\text{Quant}}(\lambda )=\frac{F_{\text{Raw}}(\lambda )G[\mu_{a}(\lambda_{x}),\mu_{s}^{\prime }(\lambda_{x})]}{R(\lambda_{m})},$$ (4)

where $\lambda_{x}$ and $\lambda_{m}$ are the excitation and emission wavelengths, respectively. Fluorescence fitting with $F_{\text{Quant}}$ is performed as described in Eq. (3), where G is a scalar function with units of (a.u./μg/mL) that relates fitted estimates of PpIX fluorescence intensity [a.u.] to PpIX concentration in units of (μg/mL), such that $C^{\mathrm{PpIX}}=(G){\mathrm{FL}}_{\text{Quant}}^{\mathrm{PpIX}}$. Fluorescence fits were performed over the wavelength range 603–721 nm, with parameters estimated in the set: (${\mathrm{FL}}^{\mathrm{PpIX}}$, ${\mathrm{FL}}^{\mathrm{PP}}$, ${\mathrm{FL}}^{\mathrm{AF}}$) from raw fluorescence, and ($C^{\mathrm{PpIX}}$, $C^{\mathrm{PP}}$, $C^{\mathrm{AF}}$) from quantitative fluorescence.

#### 2.3.3 Analysis of phantom stability

For phantoms sets considering controlled variation in surfactant content (e.g., TVF variation), the time-dependent stability of optical parameters (i.e., ${\mathrm{FL}}^{\mathrm{PpIX}}$, $\mu_{s}^{\prime }$, and BVF) were evaluated by comparing parameter estimates at a sampled time [i.e., (1, 2, 3, 4, 5) h] after preparation to the parameter estimate recovered immediately following preparation of the phantom at the (0) hour time point. For phantom sets considering controlled variation in background optical properties (e.g., LVF and BVF variation) the estimated PpIX concentration ($C^{\mathrm{PpIX}}$) was also compared with the known PpIX concentration. Linear proportionality between recovered optical parameters and the true parameter values in the phantom set was established using Pearson product-moment correlation. Optical parameter estimates were analyzed to identify outliers based on comparisons with repeat measurements within each phantom; specifically, the threshold for exclusion was specified as an absolute difference from the mean of the repeats that was more than five times the median absolute deviation of the repeats.

## 3 Results

### 3.1 PpIX Fluorescence in Nonturbid Phantoms

**Fig. 2:**
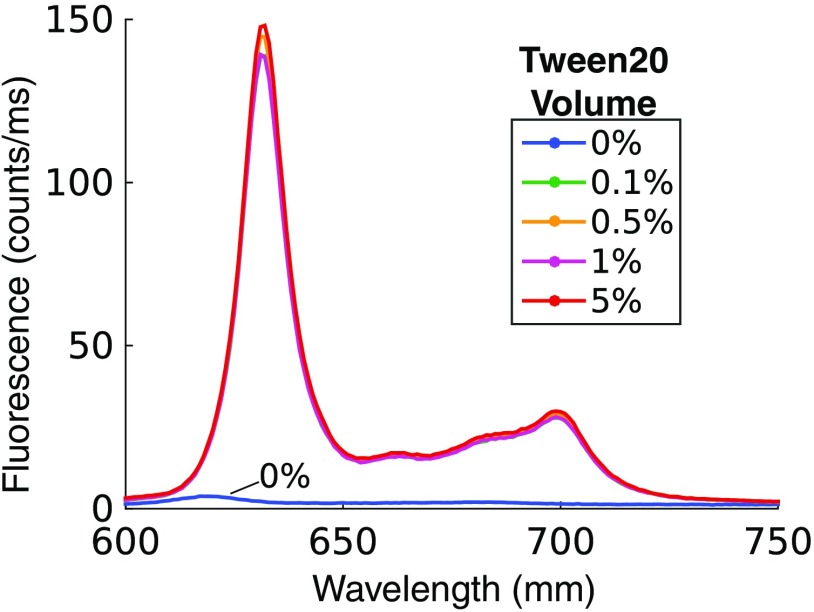
Fluorescence spectra of 1  μg/mL PpIX in PBS (nonturbid) for multiple Tween 20 concentrations.

Figure 2 shows fluorescence spectra acquired with the spectrofluorometer from a clear solution containing 1  μg/mL PpIX with various TVF. Emission spectra from samples containing surfactant show repeatable fluorescence profiles with a coefficient of variation for the peak fluorescence for all TVF of <4%. However, the sample containing no surfactant shows a strongly reduced fluorescence emission, with a reduction of 98% compared with the average of samples containing surfactant. Inspection of these data shows that in order to observe any significant PpIX fluorescence, a surfactant must be introduced to prevent aggregation. These data suggest that relatively small amounts of Tween (i.e., 0.1%) were sufficient to prevent PpIX aggregation.

### 3.2 PpIX Fluorescence in Turbid Phantoms

**Fig. 3:**
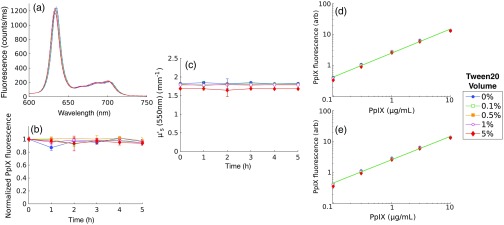
(a) Fluorescence spectra sampled in phantoms containing 1  μg/mL PpIX, 1.5% LVF, and (0, 0.1, 0.5, 1, 5)% TVF at hour 0 and hour 5. Dynamic response of optical parameters for various TVF shown for (b) fitted PpIX fluorescence, and (c) $\mu_{s}^{\prime }$ (550 nm). Estimated versus known PpIX concentrations for multiple TVF at (d) hour 0 and (e) at hour 5. Line shows linear fit to 0.1% TVF data.

Figure 3 shows PpIX fluorescence emission results collected in turbid phantoms containing 1.5% LVF and no significant background absorption. Figure 3(a) shows sample emission spectra for phantoms with 1  μg/mL PpIX and various TVF that were measured immediately following phantom preparation (i.e., time 0 h) and 5 h after preparation. These data show no observable differences in fluorescence emission for all sampled TVF for either of the sampled time points. Figure 3(b) shows fitted estimates of PpIX fluorescence at intermediate time points over the range of 0 to 5 h. Here, PpIX fluorescence was not influenced by the addition of Tween, with the maximum observed deviation of <7% among samples containing Tween volume fractions of [(0, 0.1, 0.5, 1, 5)%]. Figure 3(c) shows that estimates of $\mu_{s}^{\prime }$ (550 nm) were stable over time, with the maximum observed decrease of <1% over 5 h. The recovered value of $\mu_{s}^{\prime }$ (550 nm) was also stable in the presence of surfactant variation, with a maximum deviation of <1.5% observed for TVF ≤4%, while phantoms containing 5% TVF showed a slight decrease of <8% in $\mu_{s}^{\prime }$; such a deviation may be due either to Tween effects or to variation in the phantom making process. Figures 3(d) and 3(e) show that the estimated and known PpIX concentrations were highly correlated (r>0.99) for all TVF at 0 and 5 h.

**Fig. 4:**
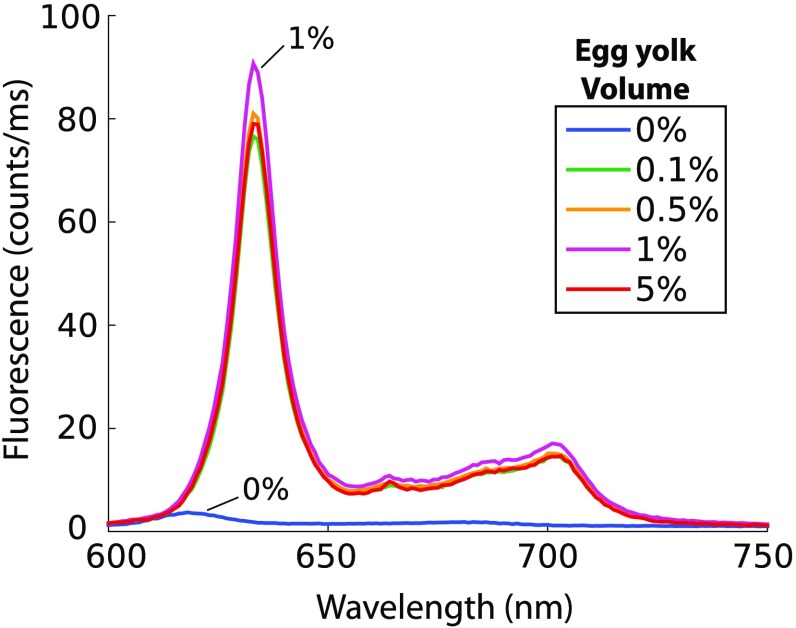
Fluorescence spectra of PpIX in a clear solution containing various concentrations of egg yolk.

The data in Fig. 3 showed that PpIX fluorescence was not influenced by the presence of surfactant, an observation that seemed to contradict the conclusion that surfactant was necessary to produce measurable PpIX fluorescence in an aqueous phantom, as shown in Fig. 2. The observed fluorescence in Intralipid phantoms with no added Tween suggested that one of the compounds in the lipid emulsion acted as a surfactant. The ingredients of the lipid emulsion included 20% soybean oil, 1.2% egg yolk phospholipids, 2.25% glycerin, and water. Egg yolk phospholipids were identified as having amphipathic properties, and it was hypothesized that this component may act as a surfactant. Figure 4 shows emission from nonturbid phantoms containing 1  μg/mL PpIX and various amounts of egg yolk, showing an average decrease in fluorescence of <95% without egg yolk. These results suggest that the egg yolk phospholipids contained within Intralipid may act as a surfactant to prevent aggregation of PpIX within an aqueous tissue phantom.

### 3.3 PpIX Fluorescence in Turbid Phantoms Containing Whole Blood

**Fig. 5:**
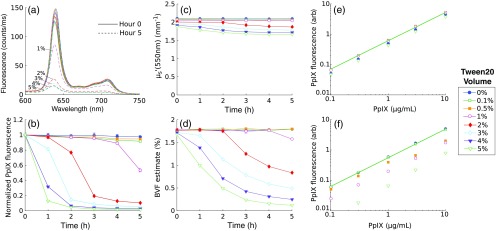
Fluorescence spectra sampled in phantoms containing 1  μg/mL PpIX, 1.5% LVF, 2% BVF, and (0, 0.1, 0.5, 1, 2, 3, 4, 5)% TVF at hour 0 and hour 5. Dynamic response of optical parameters for various TVF shown for (b) fitted PpIX fluorescence, (c) $\mu_{s}^{\prime }$ (550 nm), and (d) BVF. Estimated versus known PpIX concentrations for multiple TVF at (e) hour 0 and (f) at hour 5. Line shows linear fit to data for 0.1% TVF.

**Fig. 6:**
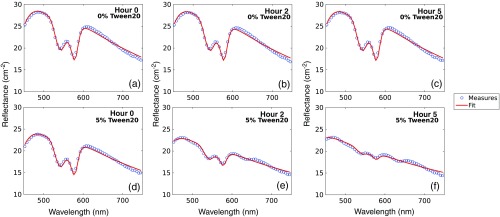
Fitted reflectance spectra from phantoms containing 1  μg/mL PpIX, 1.5% LVF, and 2% BVF at hour 0, 2, and 5, for 0% TVF [(a) through (c)] and 5% TVF [(d) through (f)].

Figure 5 shows PpIX fluorescence emission results that were collected in turbid phantoms containing scattering (1.5% LVF) and a strong background absorber (2% BVF). Figure 5(a) shows sample emission spectra for phantoms with 1  μg/mL PpIX and various TVF values at 0 and 5 h after preparation, with substantial reduction in remission intensity observed with increasing TVF values between the 0 and 5 h time points. Figure 5(b) shows the evolution of PpIX fluorescence over time for each sampled TVF; these data are for for 1  μg/mL but observed trends were similar for all PpIX concentrations sampled (data not shown). The data show that the change in fluorescence intensity with time is not linearly influenced by TVF; after 5 h the average decrease in measured fluorescence was <98% for phantoms containing 5% TVF, 47% for phantoms containing 1% TVF, and <10% in phantoms containing 0.1% and 0% TVF. Figure 5(c) shows estimates of $\mu_{s}^{\prime }$ versus time for the phantoms, with temporal variation <12% observed for all TVF, suggesting stability of the lipid induced scattering. Figure 5(d) shows estimates of BVF versus time, showing a TVF-dependent decrease in estimated BVF over time. The decreases in BVF were highly correlated (r=0.94) with reductions in PpIX fluorescence over time as shown in panel (b). These data suggest that an interaction between whole blood and Tween can complicate the stability of PpIX fluorescence over time, and that low volume fractions of surfactant (i.e., 0 to 0.1% TVF) in Intralipid yield a temporally stable fluorescence response. This hypothesis was supported by microscopic inspection of samples from phantoms obtained at the 5-h time point, revealing intact erythrocytes for 0 and 0.1% TVF phantoms, and lysed erythrocytes in the solution containing 5% Tween (data not shown). Figure 5(e) shows linear proportionality between PpIX fluorescence intensity and known PpIX concentration immediately after mixing for all TVF samples (r=0.99). Figure 5(f) shows accurate recovery of known PpIX concentration (r>0.99) for TVF ≤0.1% at 5 h after phantom preparation, but substantial TVF-dependent decreases in fluorescence intensity over time for TVF>0.1%. Figure 6 shows the spectral features of the measured and model-fitted reflectance over time for phantoms containing no Tween [(a) through (c)] and 5% TVF [(d) through (f)]. A time-dependent deterioration of the absorption signature of hemoglobin is observed in the phantom containing 5% TVF; the time-line of these spectral changes is consistent with the decrease in fluorescence and BVF estimates reported in Figures 5(b) and 5(d).

**Fig. 7:**
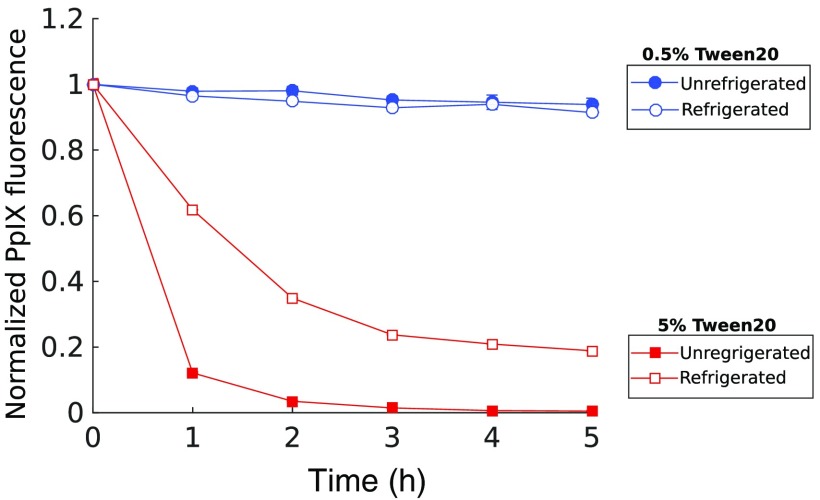
PpIX estimate over time of refrigerated and unrefrigerated phantoms for 0.5% and 5% Tween volume fraction.

The surfactant-induced degradation of fluorescence intensity over time shown in Figure 5 was observed in phantoms stored at room temperature. Figure 7 considers the comparative influence of reducing temperature by storing phantoms in a refrigeration unit. PpIX fluorescence measured in 5% TVF showed a slight reduction in the rate of fluorescence quenching for refrigerated versus unrefrigerated samples, with 38% versus 88% decrease in fluorescence at 1 h following preparation; however the cooler temperature did not yield a stable phantom, with a reduction of 81% fluorescence at the 5-h mark. Conversely, for the 0.5% TVF phantom, there was no observed difference between the refrigerated and unrefrigerated cases. These results further support the hypothesis that the origin of fluorescence variation is not due to natural decomposition of either the Intralipid or blood but is instead caused by interactions between surfactant and erythrocytes.

### 3.4 PpIX Fluorescence with Variations in Background Scattering and Absorption

**Fig. 8:**
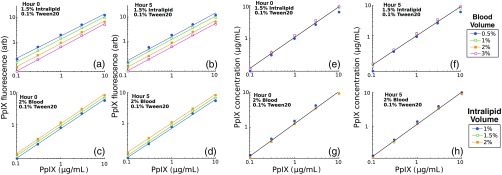
Raw PpIX fluorescence vs. concentration for multiple blood concentrations at time (a) 0 and (b) 5 h following phantom preparation. Raw PpIX fluorescence vs. concentration for multiple Intralipid concentrations at time (c) 0 and (d) 5 h following phantom preparation. Quantitative PpIX estimates versus concentrations for multiple blood concentrations at time (e) 0 and (f) 5 h following phantom preparation. Quantitative PpIX estimates versus concentration for multiple Intralipid concentrations at time (g) 0 and (h) 5 h following phantom preparation. Line in panels (e) through (h) shows linear fit to the data from all blood/intralipid volumes.

Figure 8 contains estimates of PpIX fluorescence and concentration recovered in phantoms with variations in both BVF and LVF. Figures 8(a) and 8(b) show fitted PpIX fluorescence intensity versus known PpIX concentrations for variation in BVF (range: 0.5% to 3%) at 0 and 5 h sampled time points, while Figures 8(c) and 8(d) display the same metrics for multiple LVF (range: 1% to 2%). Here, each combination of Intralipid and whole blood yielded a linear relationship between fitted PpIX fluorescence intensity and PpIX concentration, with an average Pearson product correlation coefficient of r>0.99 for each individual optical property set. The raw fluorescence data show a stratification in intensity that is due to attenuation from background optical properties, with mean absolute residuals of <34% and <13% for BVF and LVF variations, respectively. Figure 8 also shows quantitative estimates of PpIX concentration versus known concentration for variations in both BVF [(e) and (f)] and LVF [(g) and (h)] for 0- and 5-h time points. These data are corrected for the influence of background optical properties and show reduced variability with respect to variation in both BVF and LVF, with mean absolute residuals of <10% and <7%, respectively. The aggregate PpIX concentrations show a strong linear relationship between estimated and known concentration at both 0 and 5 time points (r>0.99). The observed stability in PpIX fluorescence and concentration indicates that blood and Intralipid can reliably be used as absorbing and scattering agents in optical phantoms bearing PpIX even though they are not established standards.

## 4 Discussion

The data presented in this study are used to identify a recipe for tissue-simulating optical phantoms that yield proportional and stable PpIX fluorescence emissions. Optical measurements with low TVF (i.e., ≤0.1%) presented a linear proportionality between fluorescence intensity and PpIX concentration (0.1  to 10  μg/mL) for a range of LVF (1% to 2%) and BVF (0.5% to 3%) that was stable for 5 h after mixing. Data also provided multiple insights into how surfactant influences PpIX fluorescence emission within the phantoms, including: (I) measurements showed aggregation-based quenching of PpIX fluorescence emission in aqueous nonturbid phantoms with no surfactant. (II) No difference in PpIX fluorescence intensity was observed for Intralipid diultions of (1% to 2% IVF) with or without additional surfactant, indicating that when Intralipid is used as a scattering agent, the amphipathic phospholipids contained in the lipid emulsion formulation act as a surfactant that prevents aggregation; this result was confirmed with measurements using egg yolk. (III) For phantoms containing whole blood and TVF>0.1%, we observed a temporal degradation in PpIX fluorescence intensity, with the rate of degradation dependent on surfactant concentration. These distortions were caused by an interaction between surfactant and whole blood, and this interaction was avoided by using little or no Tween (i.e., ≤0.1% volume fraction) in phantoms containing whole blood.

The optical phantoms presented in this study can be tuned to mimic the endogenous optical properties experienced in tissue. Intralipid is widely used in the field of biomedical optics as a scattering source that is relatively inexpensive, stable, and representative of the reduced scattering spectrum reported in tissue.^26^^–^^28^ This study highlights an additional advantage, that the lipid formulation contains surfactant that prevents PpIX aggregation. The use of bovine whole blood as the background absorber is well-characterized ^29^ and offers a tailored match of the absorption bands at excitation and emission to endogenous absorption in tissue. Replicating the endogenous spectral absorption characteristics of tissue across the UV–VIS wavelength range with a mixture of dyes can be difficult to achieve, with blue dyes often containing additional peaks in the visible wavelength range. The accurate recovery of quantitative PpIX concentration in the presence of variations to both blood and lipid volume fractions indicate that the absorption and scattering-based attenuation of fluorescence intensity observed in these phantoms were well described by an analytical model of light transport. Additionally, the data presented in this study showed no fluorescence distortions that could be attributable to interactions between Intralipid and bovine whole blood over a range of mixtures that replicate physiologically relevant optical properties.

This study defines a stable and useful phantom as one that returns a linear response between fluorescence intensity and the contained PpIX concentration. It is worth noting that this study does not independently measure the quantum yield of PpIX in the phantoms. While differences in the quantum yield between phantom and tissue would limit the direct use of fluorescence emission data as a standard curve to convert PpIX fluorescence intensities measured in tissue to PpIX concentration, such a conversion would likely differ by a scalar factor. The linear response of PpIX fluorescence-concentration identified for each set of optical properties suggests that changes to background scattering and absorption were not dynamically influencing the quantum yield. Therefore, these fluorescence intensity data can be used to characterize the sensitivity of raw fluorescence emission to background scattering and absorption, and an assumed quantum yield could be used to recover a linearly proportional estimate of PpIX concentration.

It should be noted that some aspects of these phantoms requires careful consideration to yield repeatable results. One consideration is that samples containing whole blood are susceptible to settling, with the blood occasionally coming out of solution and pooling at the bottom of the phantom. Gentle, but thorough, mixing of the phantom can solve this issue and it was observed that the addition of 0.1% Tween helped to keep the blood in solution. Another consideration is that accurate spectral analysis using whole blood as an absorber requires the user to measure the hematocrit of the blood to establish the concentration of total hemoglobin, which specifies the absorption spectrum. Conversely, the data indicate that the phantoms do not need to be refrigerated, with stable fluorescence response yielded over a 5-h period at ambient room temperature.

In conclusion, the present study provides data to guide the construction of tissue-simulating optical phantoms that yield a stable and repeatable relationship between PpIX fluorescence emission and PpIX concentration. The recommended recipe offers a wide range of PpIX concentrations, as well as background scattering and absorption spectra well-matched to mimic tissue. The standardization of optical phantoms bearing PpIX would make results from different systems that are investigated by different research groups directly comparable.
